# Molecular, cellular and physiological characterization of the cancer cachexia-inducing C26 colon carcinoma in mouse

**DOI:** 10.1186/1471-2407-10-363

**Published:** 2010-07-08

**Authors:** Paola Aulino, Emanuele Berardi, Veronica M Cardillo, Emanuele Rizzuto, Barbara Perniconi, Carla Ramina, Fabrizio Padula, Enrico P Spugnini, Alfonso Baldi, Fabio Faiola, Sergio Adamo, Dario Coletti

**Affiliations:** 1 Department of Histology and Medical Embryology, Sapienza University of Rome, Via Scarpa 16, 00161 Rome, Italy and Interuniversity Institute of Myology; 2 SAFU Department, Regina Elena Cancer Institute, Via delle Messi d'Oro 156, 00158 Rome, Italy; 3 Department Biochemistry, Section of Pathology, Second University of Naples, Via L. Armanni 5, 80138 Naples, Italy; 4 DVM Veterinarian chief, Health Status and Animal Welfare, Sapienza University of Rome, Via Scarpa 16, 00161 Rome, Italy

## Abstract

**Background:**

The majority of cancer patients experience dramatic weight loss, due to cachexia and consisting of skeletal muscle and fat tissue wasting. Cachexia is a negative prognostic factor, interferes with therapy and worsens the patients' quality of life by affecting muscle function. Mice bearing ectopically-implanted C26 colon carcinoma are widely used as an experimental model of cancer cachexia. As part of the search for novel clinical and basic research applications for this experimental model, we characterized novel cellular and molecular features of C26-bearing mice.

**Methods:**

A fragment of C26 tumor was subcutaneously grafted in isogenic BALB/c mice. The mass growth and proliferation rate of the tumor were analyzed. Histological and cytofluorometric analyses were used to assess cell death, ploidy and differentiation of the tumor cells. The main features of skeletal muscle atrophy, which were highlighted by immunohistochemical and electron microscopy analyses, correlated with biochemical alterations. Muscle force and resistance to fatigue were measured and analyzed as major functional deficits of the cachectic musculature.

**Results:**

We found that the C26 tumor, ectopically implanted in mice, is an undifferentiated carcinoma, which should be referred to as such and not as adenocarcinoma, a common misconception. The C26 tumor displays aneuploidy and histological features typical of transformed cells, incorporates BrdU and induces severe weight loss in the host, which is largely caused by muscle wasting. The latter appears to be due to proteasome-mediated protein degradation, which disrupts the sarcomeric structure and muscle fiber-extracellular matrix interactions. A pivotal functional deficit of cachectic muscle consists in increased fatigability, while the reported loss of tetanic force is not statistically significant following normalization for decreased muscle fiber size.

**Conclusions:**

We conclude, on the basis of the definition of cachexia, that ectopically-implanted C26 carcinoma represents a well standardized experimental model for research on cancer cachexia. We wish to point out that scientists using the C26 model to study cancer and those using the same model to study cachexia may be unaware of each other's works because they use different keywords; we present strategies to eliminate this gap and discuss the benefits of such an exchange of knowledge.

## Background

Cancer genome projects are providing complete landscapes of the mutations that exist in tumors, making it essential to bridge the gap between high-throughput sequencing information and the molecular mechanisms underlying the natural history of cancer [1]. In this regard, there is an unprecedented need for mammal models of cancer: mice with naturally occurring oncogenic mutations have provided important information regarding cancer pathogenesis; genetically engineered mice have emerged as essential tools for both mechanistic studies and drug development in cancer research; transplantation models (xenografts) have been useful in the study of metastasis and for testing potential therapies. Mouse models of colon cancer have been extensively revised by Taketo and Edelmann [2], as well as by Rosenberg and Tanaka [3].

Cancer cachexia is a muscle wasting syndrome that affects most cancer patients [4]. Cachexia is acknowledged to be a serious complication in many chronic diseases and is associated with a poor prognosis [5]. Indeed, cachexia not only affects therapy and the patients' quality of life, but is responsible for at least 20% of cancer deaths [6]. The lack of an official definition of cachexia may have led to its prevalence being underestimated, to misdiagnoses and to conflicting data being reported [5]. A major effort has recently been made to reach the following consensus definition of cachexia: a metabolic syndrome associated with underlying illness and characterized by loss of muscle, with or without loss of fat mass, frequently associated with anorexia, inflammation, insulin resistance and increased muscle protein breakdown [7].

### The genesis of the C26 colon carcinoma model

In 1975, during an effort to establish an animal colon tumor model for biological and chemotherapy studies, colon tumors were induced and transplanted in different inbred mouse strains. Four tumors survived the first transplant, which displayed a variety of histological and malignancy features. These four tumors included the colon tumor 26, described as an undifferentiated Grade IV carcinoma that metastasizes above all in the lungs [8]. In 1981, the colon tumor 26 was further characterized *in vivo *by inoculation of serial cell doses into syngeneic BALB/c mice. The C26 line was highly tumorigenic and displayed a low tendency to metastasize; C26-inoculated mice exhibited high mortality [9]. In 1990, C26-implanted mice were reported to suffer extensive carcass weight loss (40% of the control body weight), hypoglycemia and hypercorticism in the presence of unchanged food intake. C26 caused hepatic function disorders and loss of adipose and skeletal muscle tissue, thus proving to be an appropriate model for investigating the mechanisms underlying cachexia [10]. Recently, we and others have subcutaneously implanted a solid fragment of the C26 tumor, as opposed to a cell suspension of C26 cells, in the flank or dorsal region of mice [11-13]. It is noteworthy that C26-induced cachexia varies according to the inoculation site [14].

### Use of the C26 model for cancer studies

The C26 model has been used over the last three decades for research on the natural history of carcinomas and antitumor therapy. These studies range from earlier investigations exploiting C26 cells injected directly into the spleen of syngeneic mice to study the efficacy of MMP inhibitors [15], to a report showing that reduced angiopoietin availability at the tumor site hampers neoangiogenesis and thus limits tumor growth and metastases [16]. Since liver and lung metastases are the predominant cause of colorectal cancer-related mortality, cancers of the gastrointestinal tract are widely used to develop anti-tumor therapies. In this context, C26 cells were injected in mice to investigate i) the potential of CXCR3 antagonism to counteract the progression of cancer cells to target organs [17], ii) the antitumor effect of liposomal formulation of glucocorticoids [18] and iii) the effects of combined interstitial laser coagulation and doxorubicin treatment [19]. Mice either orthotopically or ectopically implanted with C26 cells were used to study the effects of several other anti-tumor agents [20,21]. Interestingly, two subclones have been isolated of C26 cells featuring differential sensitivity to 5-fluorouracil, which may represent an important determinant of drug sensitivity and treatment response [22]. While C26 cells are initially responsive to the blockade of MAP kinase pathways, they may become resistant to MAP kinase inhibitors due to K-ras activation [23]. C26 cells have also been used to demonstrate the antitumor effects of interleukin-18, interleukin-27 and the chemokine CCL21/SLC [24-26]. Further studies based on the C26 model were aimed at developing novel karyotypic analysis approaches to verify and track the origin and evolution of tumor cell lines [27].

### The C26 model for studies on cachexia and countermeasures

The C26 tumor enhances protein catabolism mediated by muscle specific ubiquitin ligases, atrogin-1/MAFbx and MuRF1 [28]. During muscle atrophy, thick, but not thin, filament components are degraded by the ubiquitin-dependent proteasome pathway [29], which is in agreement with the finding that C26 burden induces specific loss of myosin [30] and altered myosin isoform expression [31]. Thus, it has been suggested that muscle cachexia results from highly selective targeting of protein degradation [30]. In C26-bearing mice, the dystrophin complex is downregulated, a phenomenon essential for wasting, thereby highlighting a regulatory role of dystrophin in cachexia [28]. By exploiting the C26 model, we demonstrated that Peg3/PW1 and p53 participate in a positive feedback loop that regulates cachexia and stem cell numbers in skeletal muscle [32]. We also noted that cachectic muscles are enriched in stem cells with myogenic potential, though not in inflammatory cells [33].

Chemotherapeutic agents induce muscle wasting, which consequently persists in spite of tumor remission [34]. By contrast, indomethacin, ibuprofen and appetite stimulants are among treatments shown to preserve muscle mass in C26 tumor-bearing mice [35,36]. IL-6 mediates muscle wasting induced by C26, even though it is not the sole inducer of cachexia [37,38]. Indeed, it is the milieu of circulating cytokines to determine the output in terms of muscle wasting in C26-bearing mice, as indicated by the finding that IL-27 treatment rescues muscle wasting in these animals [25,37]. Myostatin negatively regulates skeletal muscle mass, though inhibition of its downstream pathways does not attenuate C26-induced cachexia, thereby suggesting that myostatin does not play a role in this context [39]. Anaerobic glycolysis in a C26 tumor is related to weight loss, while erythropoietin administration has been shown to reduce weight loss [12]. Accordingly, exercise training attenuates C26-induced muscle wasting [40]. High protein content, leucine and fish oil reduce improves functional performance in mice with cancer cachexia highlighting the relevance of dietary supplementation for cachexia [41].

Given the clinical relevance of standardizing animal models of cachexia, we performed a full characterization of the C26-bearing mice, with the aim to provide a reference for further studies on an established model of cancer which has been poorly described from the point of view of cancer-associated cachexia. We pinpointed several outputs, from organismal to molecular level, suitable for the assessment of the progression of cancer and/or cancer-associated cachexia. Here we report for the first time the rates of C26 tumor proliferation and apoptosis, a detailed description of muscle wasting in relation to muscle fiber type, ultrastructural features of the sarcomere in cachexia underlying the novel, distinguishing functional features of the wasting muscle, i.e. fatigue in the absence of loss of specific force. These features characterize, on a functional point of view, cancer cachexia from other forms of muscle atrophy, such as sarcopenia, disuse or dystrophy-associated atrophy.

## Methods

### Mice and tumor transplant

Cachexia was induced by subcutaneous grafting, using a trocar, of a 0.5 mm^3 ^fragment of colon carcinoma (C26, obtained from the National Cancer Institute) in the dorsal region of 7-week-old BALB/c mice (Charles River, Wilmington, MA). The tumor was either taken from a frozen stock or dissected from a donor mouse within 30 min of the transplant. Tumors never went through more than 15 passages *in vivo *(1 passage defined as growth from transplant to 0.5-1 g). Animals were sacrificed 3 wks following tumor implantation, unless otherwise specified. Carcass weight was calculated as total body weight devoid of tumor weight. Mice were treated in strict accordance to the guidelines of the Institutional Animal Care and Use Committee and to relevant national and European legislation, throughout the experiments.

### Flow cytometric analysis

For bromodeoxyuridine (BrdU) experiments, the mice received two injections of BrdU (50 mg/Kg body weight each) 4 hrs apart on the day before analysis [42]. A 1:25 dilution of a monoclonal antibody against BrdU (clone B44, BD, San Jose, CA) was used, followed by an Alexa488-conjugated anti-mouse antibody (Molecular Probes). DNA content was stained by incubation of the cells with a PI/RNase solution (50 μg/ml and 100 U/ml) for at least 3 hrs. Cell suspensions were analyzed with a Coulter Epics XL flow cytometer (Beckman Coulter, Fullerton, CA, USA). Gating strategy included eliminating PI-negative debris and doublets based on DNA/peak dimension ratio plotting. Samples incubated with an aspecific murine primary antibody represented negative controls. Data were analyzed with WinMDI 2.8 software (a freeware developed by J. Trotter).

### Tissue immuno-histochemical analyses

*Extensor digitorum longus *(EDL) muscles were dissected with their tendons from each control and tumor-bearing mouse, pooled and treated as described elsewhere [43]. Tumors or *tibialis anterior *muscles (TA) were frozen in liquid nitrogen-cooled isopentane, sectioned and fixed with 4% paraformaldehyde and stained with hematoxylin (H) and eosin (E) (Sigma, St. Louis, MO) following standard procedures or, alternatively, immunostained with a rabbit anti-laminin antibody (Sigma). Antibody binding was visualized by using Alexa488-conjugated goat anti-rabbit IgG (Molecular Probes) while nuclei were visualized by Hoechst staining.

Apoptosis was assayed by TUNEL (Apoptag kit, Oncor, Gaithersburg, MD), according to the supplier's instructions. Apoptotic and mitotic indexes were calculated on H&E- stained sections by counting the number of mitotic figures or TUNEL+ nuclei visible on high-power-field 40× objective in at least ten fields/sample and expressing the results as percentage of the total number of cells in the same fields.

NADH transferase staining was performed as described previously [33]. Morphometric analysis was performed on type IIb (low NADH transferase activity, glycolytic) and type I (high NADH transferase activity, oxidative) fibers separately, as described previously [44]. For each muscle, the whole muscle cross section was analyzed to calculate the average fiber size (cross-section area) by using ImageJ 1.41 (freeware developed by Dr. W. Rasband at NIH, and available at http://rsb.info.nih.gov/ij). Photomicrographs were obtained by means of an Axioskop 2 plus system (Zeiss, Oberkochen, GE) or a Leica Leitz DMRB microscope fitted with a DFC300FX camera (Leica, Wetzlar, Germany).

### RT-PCR and Western blot analysis

Total RNA was prepared from the TA muscle using Trizol Reagent (Invitrogen, Carlsbad, CA), following the manufacturer's protocol. RT-PCR and Western Blot (WB) analyses were performed using 2 μg of total reverse-transcribed RNA and 80 μg of proteins [45]. WB membranes were probed with a monoclonal antibody against ubiquitin (clone P4G7-H11, Stressgen, Ann Arbor, MI).

### Functional analysis

Functional analysis was performed according to a previously described protocol [45-47] on EDL and *Soleus *muscles. The muscles were electrically stimulated by means of a pair of electrodes, and evoked forces continuously acquired. To evoke tetanic force (maximal force), muscles were stimulated with two trains of 0.1 ms pulses (180 Hz pulses for 0.4 s). *Specific force *was calculated by dividing the tetanic force by the cross-sectional area of each muscle [46]. Muscles were subjected to a further series of closer trains of pulses (0.4 s train of 120 Hz pulses) to induce isometric fatigue. During the fatigue stimulation, we measured the ability of the muscle to resist to repeated stimulations by calculating the time required to halve the value of its own maximum force (*fatigue time*).

## Results

### C26 tumor histopathology

**Figure 1:**
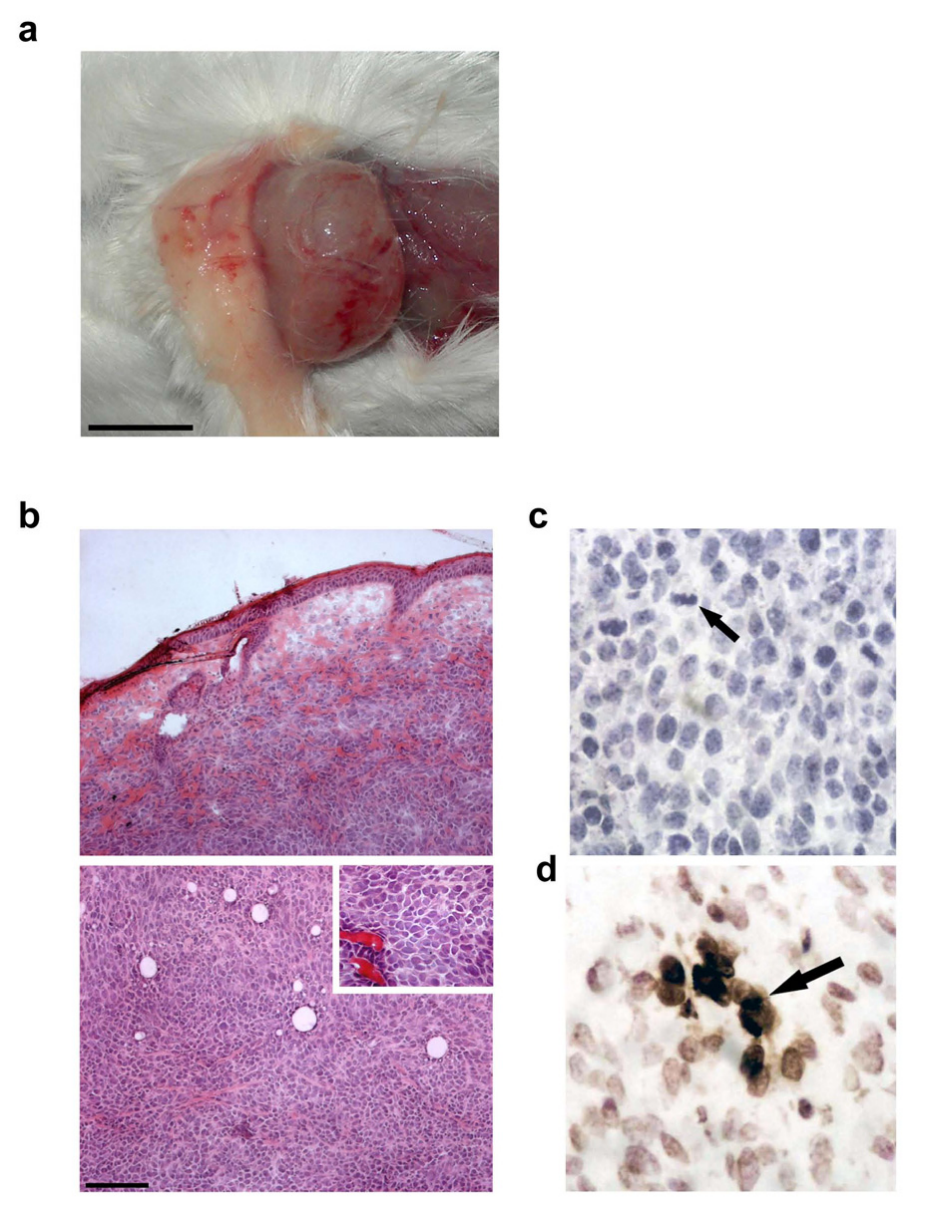
**Morphological and histological features of C26 tumor**. a) Exposed C26 tumor, three weeks following subcutaneous transplant of a 0.5 mm^3 ^tumor fragment, has grown, becoming a spheroid measuring 0.5-1 cm in diameter. The tumor mass is well-defined and vascularized. Bar 5 mm. b) Photomicrographs of H&E-stained tumor cryosections. The tumor was collected three weeks following tumor transplant for histological analysis. *Top*, peripheral region showing tumor encapsulation and, *bottom*, inner region showing anaplastic appearance and lack of a necrotic core. *Inset*, cell morphology and vascularization at higher magnification. Bar = 100 μm c) Mitoses (*arrow*) were evaluated on C26 tumor sections, based on cell morphology identified as typical of prophase to telophase, and expressed as percentage of the cells counted in 10 randomly chosen fields for each tumor. An average mitotic index of 5 ± 2% (mean ± SEM) was calculated from data derived by four independent experiments (n = 8). d) TUNEL assay on C26 tumor sections of the same samples used for the mitotic index (the *arrow *points to a TUNEL+ cell) yielded an apoptotic index of 9 ± 3% (mean ± SEM).

We subcutaneously implanted a solid fragment of about 0.5 mm^3 ^of C26 colon carcinoma in the dorsal region of mice. During the first week post-transplant, it was possible to locate the tumor by palpation. During the second week post-transplant, it was possible to see the site of tumor implant as a protrusion of the skin. During the third week post-transplant, tumor growth was evident, it being possible to see a mass underneath the skin; the mass occasionally ulcerated, causing open wounds. When surgically exposed, the C26 tumor was large, stiff and roughly spherical in shape (Figure 1a). The tumor was vascularized; it displayed a necrotic core when the diameter significantly exceeded 1 cm, the mass in this case weighing more than 1 g (data not shown).

The histological analysis revealed that the C26 is a partially encapsulated, anaplastic carcinoma. The cells varied in size, as did the nuclear/cytoplasm ratio. The degree of vascularization was good for an ectopically-located tumor, it being sufficient to sustain tumor growth and survival. (Figure 1b). The mitotic and apoptotic indexes (Figure 1c and 1d) were 5 ± 2% and 9 ± 3%, respectively. Histochemical analysis showed the absence of inflammatory infiltrate within the tumor mass. In particular CD3+ and CD8+ leukocytes, as well as macrophages, were undetectable in, or immediately around, the tumor (data not shown). Expression of Peg3, a growth-inhibitory imprinted gene which is frequently down-regulated in cancer, was absent (data not shown).

### Characterization of C26 tumor growth

**Figure 2:**
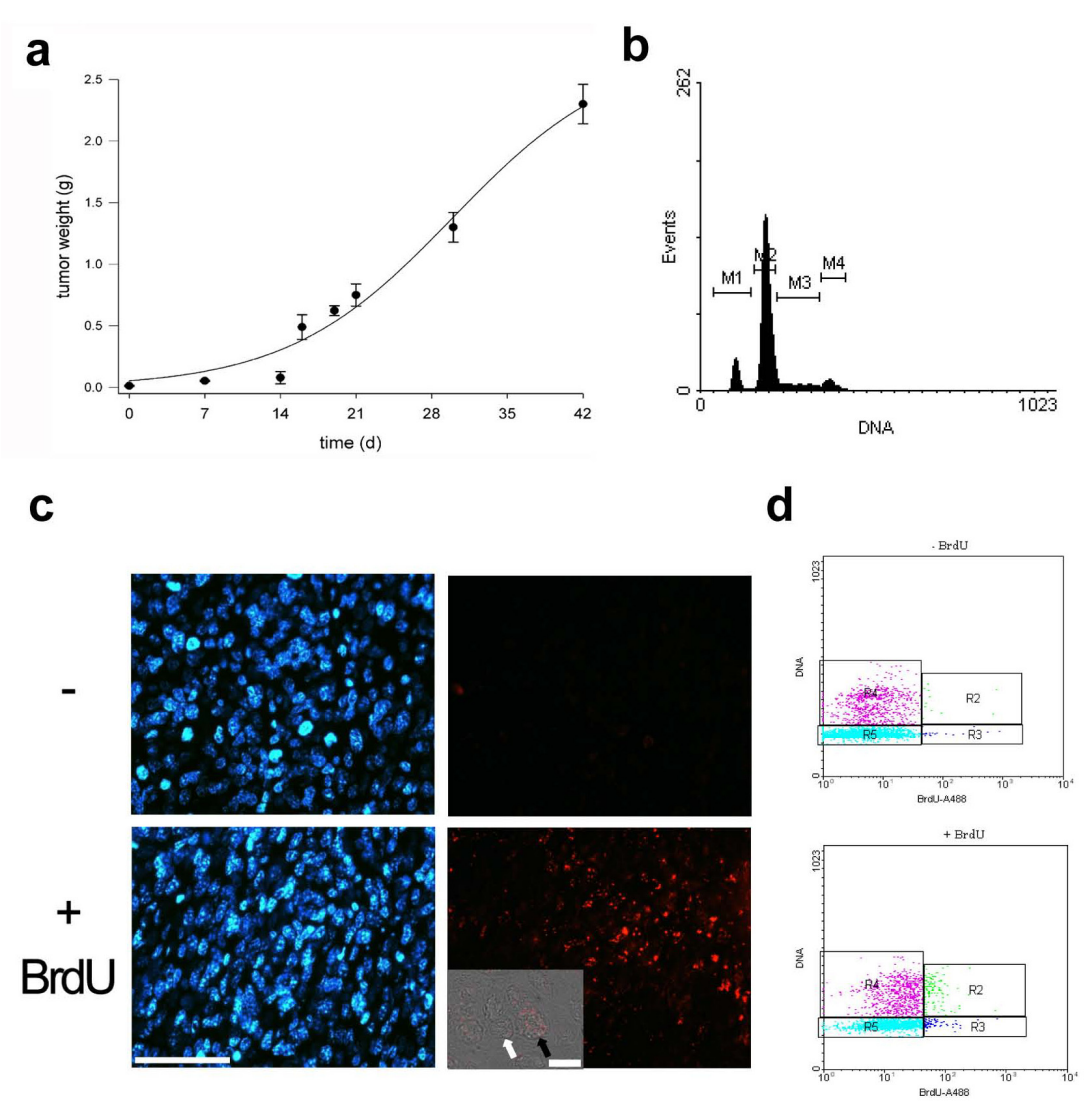
**C26 tumor proliferation**. a) Tumor mass kinetics, measured upon tumor explant at the indicated times. After a lag time of about two weeks, tumor growth is linear and results in a mass of considerable size (>2 g, i.e. about 10% of the body weight). The mean ± SEM of at least three independent experiments performed in triplicate is shown (for each data point, the number of replicates, "n", is: 9 < n < 22). b) The DNA profile by flow cytometry of PI-labeled cells, at 3 weeks following transplantation, shows: a significant sub-population of cells in the S phase (M3), which indicates that tumor cells are actively proliferating; a haploid, sub-G1 peak (M1) that does not resemble apoptotic cells/debris; lack of polyploidism; M2 and M4 indicate the G1 and G2 sub-populations, respectively. c) BrdU incorporation (*red*) was detected in the tumor cells at 3 weeks following transplantation, the day after IP injections of two doses (50 mg/Kg of body weight) of BrdU 4 h apart (*+ BrdU*) though not in the sham-injected mice (-). Nuclei are counterstained with Hoechst (*blue*). Bar = 100 μm. *Inset*, confocal phase contrast image merged with the red fluorescence reveals the presence of BrdU+ and negative nuclei (*black *and *white arrow*, respectively). Bar = 10 μm d) Flow cytometric analysis of BrdU incorporation in cells enzymatically extracted from C26 tumors at 3 weeks following transplantation. BrdU+ cells were plotted against PI labeling (*bottom*) and compared to C26 cells from sham-injected mice. Cells above background are in R2 and R3.

The tumor growth kinetics revealed a lag phase for the first two weeks after transplant, followed by a growth phase that gave rise to tumors larger than 2 g (Figure 2a). We noted that growth kinetics gradually became slower with the progression of the tumor passages *in vivo *(data not shown). Three weeks following transplant, flow cytometric analysis of PI-labeled tumor cells revealed the presence of a significant sub-population of cells in the S phase (15 ± 1%); in addition, no polyploidism was found; we instead noticed the presence of a hypodiploid peak (Figure 2b). BrdU incorporation, corresponding to 9 ± 2% of the cells by flow cytometric analysis (Figure 2d), was detected in cell nuclei by immuno-fluorescence (Figure 2c). By plotting BrdU positive cells versus PI staining, we noted several BrdU+ cells in the G1 phase, which indicates that a significant fraction of the cell population proceeded through an entire cell cycle. Although we did not systematically perform complete autopsies of the sacrificed animals, we encountered only one case of metastasis in the liver 50 days after tumor transplant, which suggests a low metastatic potential.

### Host systemic response to the C26 tumor

**Figure 3:**
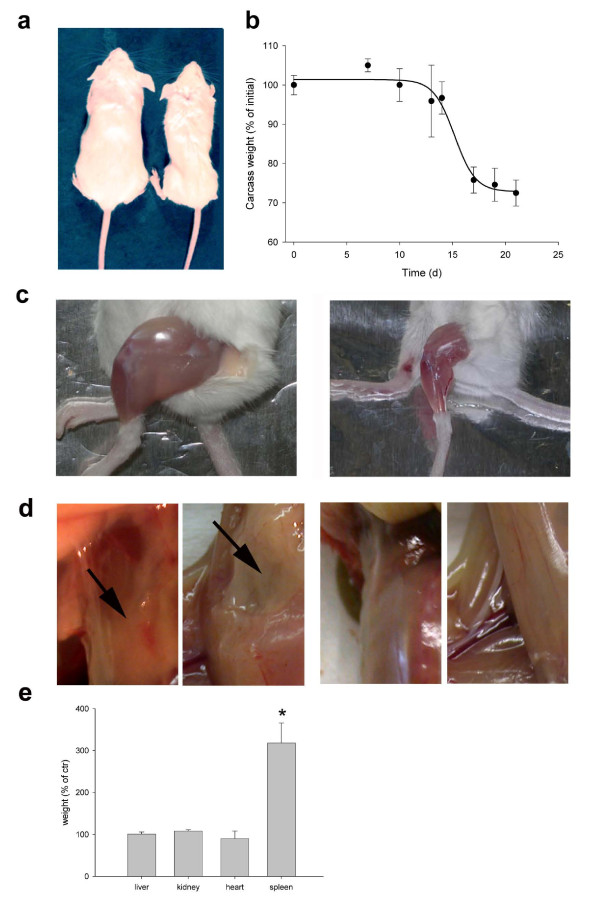
**C26-induced cachexia**. a) Gross appearance of control (*left*) compared with tumor-bearing mice (*right*) at 3 weeks following tumor implant. b) Carcass weight loss, defined as the total weight minus the tumor weight at given time points and reported as a percentage of the initial weight of each mouse. Non-significant weight loss is observed for up to two weeks of tumor burden, followed by 30% weight loss in the third week. c) If compared with a healthy mouse (*left*), the hindlimb of a tumor-bearing mouse (*right*) is severely atrophied at 3 weeks following transplantation. d) Dorsal and abdominal view of subcutaneous fat pads in control *(left panels, arrows) *and C26-bearing mice *(right panels) *at 3 weeks following transplantation. The subcutaneous fat is virtually absent in cachexia. e) The weight of several internal organs is unaffected 3 weeks following tumor transplant, while the spleen is hypertrophic. p > 0.01 by Student's *t *test if compared with the weight of matching organs from control animals. The mean ± SEM of two experiments performed in triplicate is shown.

C26 tumor induced the death of 90% of the mice within 32 days from the transplant, with an average survival time of 25 days (data not shown). The hallmark of the host response to tumor load was cachexia (Figure 3a). Tumor-bearing mice appeared emaciated and had disheveled fur (Figure 3a). Body weight loss was unaffected in the first two weeks following tumor transplant, but was catastrophic in the third week, when tumor-bearing mice reached a 30% weight loss plateau (Figure 3b). Body weight loss was largely accounted for by muscle wasting (as shown in Figure 3c) accompanied by disappearance of fat pads (Figure 3d). Although we did not perform a systematic analysis, we observed that numerous skeletal muscles throughout the body were affected by muscle wasting, as shown by decreased muscle mass (Table 1). Two-way ANOVA demonstrated a significant effect on muscle mass induced by the presence of the tumor (i.e. *bona fide *cancer-induced muscle wasting). ANOVA indicated also no interaction between the two variables, i.e. muscle type and presence of the tumor, in affecting muscle mass. In fact, the muscles we analyzed displayed a similar degree of wasting, regardless of their intrinsic differences in fiber number and physiological properties (Table 1). Skeletal muscle and fat tissue appeared to be affected most by the presence of C26 tumor, there being several organs that did not waste to a similar degree (Figure 3e). We noted splenomegaly, characterized by a significant, 3-fold increase in the spleen weight of tumor-bearing mice (Figure 3d).

**Table 1:** C26 induces muscle wasting

TREATMENT	MUSCLE		
	
	*Soleus*	EDL	*Tibialis*
Control (weight, g)	6.3 ± 0.2	12.5 ± 0.4	42.7 ± 2.4
C26 (weight, g)	4.9 ± 0.1 *	9.7 ± 0.3 *	30.5 ± 0.9 *
C26 (weight, % of control)	77%	77%	71%

The indicated muscles were dissected from control or tumor-bearing (C26) mice three weeks following tumor transplant and their wet mass evaluated. Reported is the mean ± SEM of at least three independent experiments (for each data point, the number of replicates, "n", is: 4 < n < 10). Two-way ANOVA (F = 92.3, p < 0.0001 between rows; F = 534.9, p < 0.0001 between columns) indicates a statistically significant effect on muscle mass, independently of muscle type, induced by the presence of the tumor. * = p < 0.01 versus matching control by Student's *t *test.

### C26 tumor-induced muscle fiber atrophy

**Figure 4:**
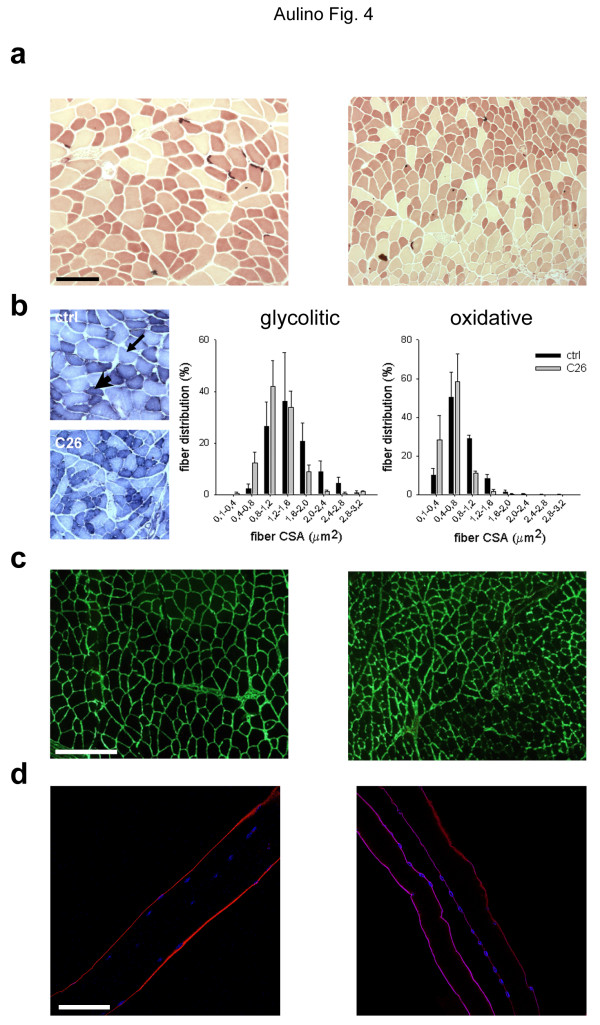
**Muscle fiber atrophy in C26-bearing mice**. a) Esterase staining of TA cross-sections from control (*left*) and tumor-bearing (*right*) mice, three weeks following transplant. Fiber atrophy is evident in different fiber types in the absence of lysosome accumulation. The intensely stained neuromuscular junctions are also visible. Bar = 50 μm. b) NADH-transferase staining can be used to differentially analyze larger, glycolytic fibers (*arrow*) and smaller, oxidative fibers (*arrowhead*), in TA cross-sections from control (*top*) and tumor-bearing (*bottom*) mice. Intermediate fibers are also visible. The fiber cross-sectional area (*CSA*) was measured, and the distribution is shown for both glycolytic and slow fibers, in control (*black bars*) and C26-bearing (*gray bars*) mice. The average size ± SEM of each fiber size class calculated from replicate experiments is shown, along with the medians of each distribution. C26-induced fiber atrophy is detectable in both fiber types. c) Immunostaining for laminin (*green*) on TA cross-sections from control (*left*) and tumor-bearing (*right*) mice, three weeks following transplant, showing alterations in the basement membrane. Bar = 100 μm. d) Immunostaining for laminin (*red*) performed on enzymatically isolated fibers, obtained from EDL of control (*left*) and tumor-bearing (*right*) mice, and longitudinally depicted by confocal microscopy. Nuclei are counterstained with TO-PRO and pseudo-colored in blue. Bar = 30 μm.

While muscle fibers being the bulk element of the musculature, whole muscle mass is affected by several tissues, which are intermingled with skeletal muscle fibers. In evaluating muscle wasting it is therefore important to assess muscle fiber specific events. Thus, we analyzed the muscle fiber specific response to tumor burden by a combination of immunohistochemical and morphometric approaches. The lysosomal proteolytic system is stimulated in adult muscles in a variety of pathological conditions; lysosome accumulation in the fibers was not, however, observed in cancer cachexia by aspecific esterase staining (Figure 4a); nonetheless, we did note that tumor load induced marked muscle fiber atrophy. To quantify this phenomenon, we performed a morphometric evaluation of a muscle fiber cross-sectional area on subpopulations of fibers with different biochemical properties, based on NADH-transferase. The latter identifies oxidative, glycolytic and intermediate fibers according to their mitochondrial content and oxidative capacity (Figure 4b). We found that the C26 tumor induced a shift in both glycolytic and oxidative fibers toward smaller cross-sectional areas (Figure 4b). Accordingly, muscle fiber atrophy was apparent when we performed immunostaining for laminin, an important component of the extracellular matrix (i.e. the basement membrane) that individually surrounds the fibers (Figure 4c). We noted that laminin staining was blurred in muscles from tumor-bearing mice, suggesting the occurrence of basement membrane disorganization (Figure 4c). The enzymatic isolation of single fibers from the musculature of mice is an approach commonly used to study muscle fibers surrounded by their basement membrane. We applied this approach to cachectic muscles; the observation of remarkable fiber shrinkage in cachexia confirmed our previous findings (Figure 4d).

### Ultrastructural alterations of myofilaments in tumor-bearing mice

**Figure 5:**
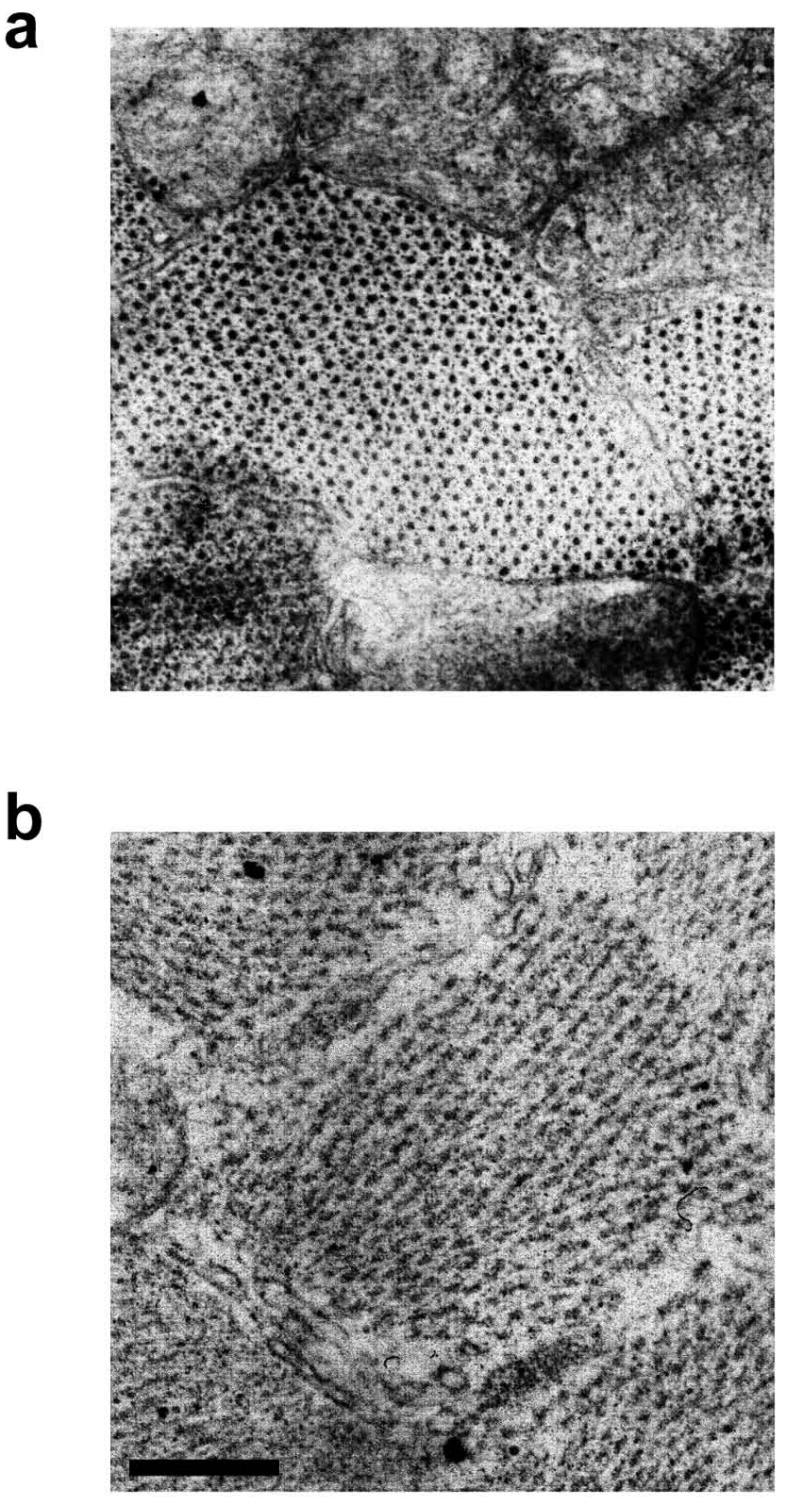
**Ultrastructural characterization of sarcomeres in ultrathin cross-sections of TA from control (*top*) and tumor-bearing (*bottom*) mice observed by transmission EM**. A disarrangement of the myofilament hexagonal organization at the level of the A-band is observed upon tumor burden. Bar = 250 nm.

Studies have shown that the myofilament protein content in atrophic muscles is reduced and the sarcomere alignment altered, as seen in longitudinal sections. We thus investigated the sarcomeric architecture in ultrathin cross-sections from control and tumor-bearing mice. At the level of the A-band, normal sarcomeres appeared full of both thick and thin filaments, the latter in the typical hexagonal formation surrounding each myosin filament (Figure 5a). We found that most of the sarcomeres in cachectic muscle were disrupted, that both types of filament were poorly defined, and that while the cisternae of the sarcoplasmic reticulum were still present, the sarcomeric perimeter was less clearly delimited (Figure 5b).

### Muscle wasting associated with increased protein degradation and loss of function

**Figure 6:**
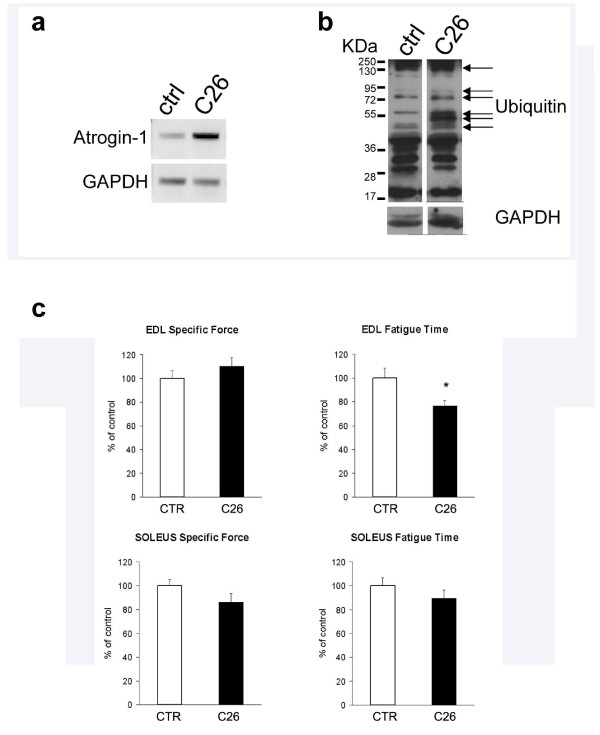
**Protein ubiquitination and fatigue induced by C26**. RT-PCR (a) and WB (b) analyses show the upregulation of E3 ligase (Atrogin-1)-mediated protein ubiquitination in the TA of tumor-bearing mice (*C26*). GAPDH is shown as loading control. c) The functional analysis of the EDL from control (*open bar*) and C26-bearing (*solid bar*) mice shows unaltered specific force (i.e. tetanic force normalized by muscle mass) and reduced fatigue time (i.e. time required to halve titanic force upon repeated stimuli) in cachectic muscles. p > 0.05 by Student's *t *test. The mean ± SEM of two experiments performed in triplicate is shown.

As proteins are the main components of bulk muscle mass, we investigated protein degradation in the musculature upon tumor burden. We noted a significant up-regulation of muscle specific E3 ubiquitin ligase Atrogin-1 expression in tumor-bearing mice, suggesting the involvement of proteasome-mediated protein degradation in cachectic muscles (Figure 6a). WB analysis of muscle extracts revealed that protein ubiquitination was qualitatively and quantitatively affected by C26 tumor (Figure 6b). These results indicate a general upregulation of protein degradation phenomena induced by tumor burden.

Muscle fiber atrophy and dismantling of sarcomeric proteins typically lead to impaired muscle performance. We evaluated muscle force and fatigue of EDL and *Soleus *muscles in control and tumor-bearing mice and found that cachectic muscle displays a lower maximal force than control muscle (data not shown), which confirms previous findings. C26 tumor did not affect the specific force, i.e. normalized by muscle size, of either muscle, but it significantly reduced the fatigue time of the EDL, thereby indicating that C26 negatively affects muscle function, mostly by diminishing resistance to fatigue (Figure 6c). When we investigated the fatigue time of the *Soleus *muscle, which has a different fiber composition from the EDL, being enriched in type I fibers, we did not notice a significant decline in muscle performance upon tumor-burden. These findings highlight heterogeneity in the functional performance of different muscles in response to tumor-burden.

## Discussion

In 2002, there were approximately one million new cases of colon cancer worldwide, making it one of the leading causes of cancer death; moreover, its prevalence was increasing in some countries [48]. The clinical relevance of colon cancer led to an unparalleled experimental use of animal models. Mice bearing the C26 colon carcinoma represent an established murine model of cancer [8,9]. Cachexia is associated with most cancers, including the murine C26 colon carcinoma [10]. Consequently, there is a striking discrepancy between the 188 papers yielded by the query "C26 AND cancer" and the 8 papers yielded by the query "C26 AND cachexia" in the NCBI's PubMed system, the most widely used method for accessing MEDLINE. Noticeably, the search using "C26 AND cancer" does not retrieve very important works on cancer cachexia that exploited the C26 model [28,32]. PubMed employs a Boolean search strategy, which suffers, among other shortcomings, from differences in term usage between searchers and indexers [49]. It is apparent that the communities of scientists exploiting the C26 model to study either cancer or cachexia are not aware of each other's works, and this may have deleterious consequences for the progress of integrative medicine applied to a complex syndrome associated with underlying illness. We suggest that "C26" be included among the keywords whenever work is conducted on this experimental model to provide adequate visibility.

The definition of the C26 tumor as adenocarcinoma is a major mistake in terminology [10,12,35]. We have confirmed that the C26 cells, originally obtained from a colon carcinoma, when ectopically implanted in mice form an undifferentiated carcinoma, which should thus be referred to as such. This tumor has a growth rate comparable to that of other carcinomas in rodents [50], with the mass growing to a significant size that corresponds to 10% of the body weight at 40 days. Such a large tumor mass is inconceivable in the clinical setting. However, it is worth noting that we observe a significant effect on both body weight loss (this work) and muscle fiber atrophy [33] far before the tumor reaches such a significant size. In particular, we note that during the lag phase of tumor growth, i.e. within the first two weeks following tumor transplantation, the presence of the tumor already affects fiber size [33]. At day 16 (i.e. at the onset of tumor massive growth) body weight loss is already significant and has reached a plateau. While body weight negatively correlates with tumor mass in some animal models, such as the MAC-16 -bearing mice [51], this correlation is not straightforward and depends on the type of tumor. Lung carcinoma-bearing mice do not loose weight during tumor growth while sarcoma-bearing mice loose more than 10% of their initial weight in the same time frame [52]. This is in agreement with an independent report showing that sarcoma-bearing rats display a catastrophic weight loss at 14 days following tumor transplant, when the latter has not significantly grown yet [53]. The fact the maximal weight loss precedes the maximal tumor growth is a useful feature since cachexia may thus be observed in the absence of significant disturbing factors, such as a relevant tumor mass. This phenomenon also indicates a non linear relation between tumor size and cachectic effects, highlighting the complexity of the underlying mechanisms. We observed relatively low standard deviations associated to any given data point of the tumor growth kinetics, even during the time lapse corresponding to the fastest tumor growth, which mirrors a good reproducibility of the experimental procedure. We inject a solid fragment (of standard dimensions) of the tumor rather than a cell suspension. We speculate that, with our approach, all the tumor cells remain in place and are exposed to the same niche, thus leading to a highly reproducible output. This is a very important issue, given the relevance of standardization in animal models of cancer-cachexia.

In keeping with tumor cell DNA distribution, C26 cells are not polyploid, but display a hypodiploid peak that is not accounted for by apoptosis. Carcinomas have been reported to be associated with hypodiploid cells, whose presence may represent a poor prognosis factor [54,55]. The growth-inhibitory imprinted gene Peg3 is not expressed by C26 cells, which is highly relevant since loss of Peg3 expression through promoter methylation, loss of heterozygosity and other mechanisms may stimulate clonogenic growth and contribute to the pathogenesis of a number of cancers [56,57]. The results regarding the metastatic potential of C26 are controversial [8,9]. In our studies, the low incidence of metastases and the time required for them to occur are in agreement with Sato et al.'s work and suggest that the C26 model may be exploited for studies on metastases-free tumors.

We found that the host response to C26 tumor burden includes splenomegaly, another controversial result [8-10]. Splenomegaly is a marker of tumor progression that is associated with leukemoid reaction [58,59]. Few studies have addressed the interactions between inflammatory cells and skeletal muscle in cachexia. We found that the number of leukocytes, neutrophils and macrophages does not increase, but may on the contrary be depleted in the endomysium of tumor-bearing mice [33]. We thus believe that the muscle damage observed in cancer cachexia [28] is not attributable to leukocytes, neutrophils and macrophages, even though inflammatory cells do induce muscle damage and regeneration in different contexts [60]. On the contrary, the partial cancer-associated immuno-depression displayed by C26-tumor bearing mice may be relevant to cachexia, since a role for immune cells in protection from cachexia has been reported in a different experimental setting [61]. These findings are in agreement with the evidence that treatments leading to an improved immune competence are beneficial against cancer cachexia [62].

The hallmark of the host response to tumor burden is cachexia [10]. Wasting is a direct effect of circulating cytokines on skeletal muscle metabolism and cannot be ascribed to cancer-associated anorexia, since food intake by C26-bearing mice is not significantly different from that of controls [28]. Nonetheless, we and others observed a significant loss of both fat and lean mass induced by the presence of a tumor. We observed that muscle wasting is associated to increased protein degradation, as shown by increased protein ubiquitination in muscles from C26-bearing mice. Cachexia, sarcopenia, and disuse atrophy are wasting conditions characterized by loss of muscle mass. These conditions result in different metabolic adaptations: increased rate of protein degradation in cachexia, as opposed to unchanged protein degradation in inactivity or sarcopenia [63]. The loss of both adipose and muscle tissue is a specific feature of cachexia, making it possible to further distinguish cachexia from sarcopenia, which is characterized by loss of lean but not fat mass. Taken together, these features allow a clear discrimination among different wasting conditions and indicate that cachexia is distinct from sarcopenia and from disuse. Accordingly, this concept has been included in the recently released consensus definition of cachexia [7].

Since loss of muscle mass is a hallmark of muscle wasting, we have measured the mass of several muscles, namely the *Soleus*, the EDL and the *Tibialis*. These muscles differ insomuch as they have very different sizes and functions, while being all anatomically located in the distal extremity of the lower limbs. Differential functions are mirrored by a different fiber type composition and oxidative metabolism, with the *Soleus *and EDL being predominantly composed of slow (oxidative) and fast (glycolytic) fibers, respectively; the *Tibialis *has a mixed fiber population. We showed that all the muscle analyzed were severely cachectic. Two-way ANOVA demonstrated that the C26 tumor significantly affects the muscle mass irrespectively of its initial size and type. We therefore conclude that muscle wasting appears to be a generalized response to tumor-burden, even though we cannot exclude that some muscles are spared (which would be demonstrated only by a systematic analysis on virtually the entire musculature). This is a novel, relevant finding; however, it does not imply that all muscles are equal with regard to all responses: for instance, both *Soleus *and EDL become cachectic in the presence of the C26-tumor but they do not have superimposable functional deficits (discussed below).

Muscle wasting is due to muscle fiber atrophy. The decline in muscle mass observed in both *Soleus *and EDL in spite of the differences in fiber type composition suggested that fiber atrophy is not fiber-type dependent. To directly address this issue, we measured muscle fiber CSA in two different fiber populations (oxidative and glycolytic fibers identified by NADH-transferase staining) in the same muscle, namely the EDL. We observed atrophy of both glycolytic and oxidative fibers, the latter previously reported to be resistant to cachexia [28], an observation based on the assessment of fiber diameter rather than on that of fiber cross-sectional area, as ours instead was. The latter method may be more sensitive to minor changes that would otherwise escape detection. Our findings that the tumor induces a significant decrease of the *Soleus *muscle mass and of the oxidative fibers in the EDL indicate that, irrespectively of muscle type, the C26 tumor does affect a fiber type generally considered "more resistant" to cachexia.

Proteasome-mediated degradation of ubiquitinated proteins leads to sarcomere dismantling [29]. Therefore, we investigated the structure of the sarcomeres in cross-section at the ultrastructural level. We showed that in cachexia disorganization occurs of the otherwise highly organized array of actin and myosin myofilaments. While the selective targeting of myosin heavy chain for degradation in cancer cachexia was recently reported [30], this is the first direct demonstration of a deficit in the contractile system of cachectic muscles. In addition to protease- and proteasome-mediated digestion, autophagy plays a significant role in muscle fiber atrophy [64], though we did not notice an increase in the number of lysosomes in cachectic muscle fibers by staining for esterase activity. This does not formally exclude a relevant role of autophagy in C26-induced muscle wasting and this issue needs further investigation. Alterations in the dystrophin complex that anchors muscle fibers to the basement membrane have been reported to occur in C26-induced cachexia [28]; this may explain the blurred appearance of laminin, one of the main components of the basement membrane. In fact, cachectic fibers show an irregular surface and a general deregulation of the cell-matrix interactions *in vivo *[28]. Dystrophinopathies in colorectal cancer patients have also been reported [28] (Zampieri S. et al., 2010 Spring Padua Muscle days, Terme Euganee, Padua, Italy, april 22-24, 2010: p. 69; in press; online on http://www.bio.unipd.it/bam/). These acquired myopathies are the earliest muscle markers of cachexia observed to date, since they occur before the onset of cancer-associated cachexia [65,66]. For all the above, we think that it is very important to assess the status of the sarcolemmal proteins, and/or of the proteins of the basement membrane connected to the sarcolemma, in a cancer cachexia study and that this should be a routine procedure. We have shown that both p53 and Peg3/PW1 are expressed in the musculature and mediate muscle atrophy [32]. These two factors are also regulators of myogenic differentiation *in vitro *and muscle regeneration in response to cytokines [13,32,44,45,67]. Since dystrophin downregulation renders cachectic muscle particularly fragile [28], a reduced regenerative capacity may cause muscle wasting. From a clinical point of view, muscle weakness and fatigue are among the leading causes of distress in cachexia. Although maximal tetanic force is reduced by C26 [68], this reduction is not substantial when force is normalized by a muscle mass index, yielding the so-called specific force. This suggests that the drop in maximal force is merely due to muscle wasting and not to alterations in the intrinsic contractile properties of the myofibers. We have shown that the EDL muscle has a 25% decrease in the fatigue time as compared to the healthy control. Thus, we propose that muscle fatigability, rather than muscle force, is a hallmark of cachectic muscle functional deficit. Interestingly, the *Soleus *muscle showed a smaller, non significant decline in the fatigue time, indicating that resistance of this muscle is spared in cachexia. This occurs in spite of the fact that the *Soleus *is atrophic in cachexia. This phenomenon highlights the heterogeneity of the muscle responses to cancer and the importance of addressing multiple parameters for different muscles to fully characterize cachexia *in vivo*.

## Conclusions

The current consensus definition for cachexia - "[...] a complex metabolic syndrome associated with underlying illness and characterized by loss of muscle with or without loss of fat mass [...]" [7]- translates into the following diagnostic criteria: weight loss of at least 5% in 12 months in the presence of illness, and at least three of the following features: decreased muscle strength; fatigue; anorexia; low fat-free mass index; abnormal biochemistry (e.g. inflammatory/protein degradation markers). While this definition has not been tested in epidemiological or clinical studies, a consensus operational definition provides an opportunity for increased research. C26 carcinoma-bearing mice display all these features with the exception of anorexia, with the caveat that a more severe (about 20%) weight loss is to be expected in mice, compared to humans. We conclude that the C26 carcinoma ectopically implanted into BALB/c mice represents a good experimental model for research on both cachexia and underlying cancer. Our work also pinpoints several biochemical, cellular and physiological outputs that can be used in a standardized manner to assess muscle wasting and the underlying tumor growth in studies aimed to demonstrate the effects of pharmacological or genetic interventions against cancer cachexia.

## Abbreviations

BrdU: 5-bromo-2'-deoxyuridine; C26: colon carcinoma C26; CSA: Cross Sectional Area; EDL: Extensor Digitorum Longus; CXCR3: CXC Chemokine Receptor 3; H&E: Hematoxylin and Eosin; IL-6: interleukin-6; MAP: Mitogen-Activated Protein; MMP: Matrix MetalloProteinase; NADH: Nicotinamide Adenine Dinucleotide: reduced; Peg3/PW1: Paternally Expressed Gene 3; PI: propidium iodide; RT-PCR: Reverse-Transcriptase Polymerase Chain Reaction; SEM: Standard Error of the Mean; TA: *Tibialis *Anterior; TUNEL: Terminal dUTP nick end labeling; WB: Western Blot.

## Competing interests

The authors declare that they have no competing interests.

## Authors' contributions

PA, EB and VC, conception, design and execution of experiments; ER, physiology experiments; BP, contributing important intellectual content; CR and FP, data collection; EPS, AB and FF, histopathology of the tumor and major intellectual contribution; SA, concept, design and editorial support, research fund collection; DC, conception and design, data collection and analysis, figure and manuscript preparation. All authors read and approved the final manuscript.

## Pre-publication history

The pre-publication history for this paper can be accessed here:

http://www.biomedcentral.com/1471-2407/10/363/prepub
